# *Sutherlandia frutescens* Ethanol Extracts Inhibit Oxidative Stress and Inflammatory Responses in Neurons and Microglial Cells

**DOI:** 10.1371/journal.pone.0089748

**Published:** 2014-02-25

**Authors:** Jinghua Jiang, Dennis Y. Chuang, Yijia Zong, Jayleenkumar Patel, Korey Brownstein, Wei Lei, Chi-Hua Lu, Agnes Simonyi, Zezong Gu, Jiankun Cui, George E. Rottinghaus, Kevin L. Fritsche, Dennis B. Lubahn, William R. Folk, Grace Y. Sun

**Affiliations:** 1 Department of Biochemistry, University of Missouri, Columbia, Missouri, United States of America; 2 Interdisciplinary Neuroscience Program, University of Missouri, Columbia, Missouri, United States of America; 3 Division of Animal Sciences, University of Missouri, Columbia, Missouri, United States of America; 4 Center for Translational Neuroscience, University of Missouri, Columbia, Missouri, United States of America; 5 Department of Pathology and Anatomical Sciences, University of Missouri, Columbia, Missouri, United States of America; 6 Center for Botanical Interaction Studies, University of Missouri, Columbia, Missouri, United States of America; Massachusetts General Hospital/Harvard Medical School, United States of America

## Abstract

*Sutherlandia frutescens* (L.) R.Br. (SF) is a medicinal plant indigenous to southern Africa and used in folk and contemporary remedies for stress, chronic diseases, cancer, and HIV/AIDS. While previous studies have focused on physiological effects of SF on cellular and systemic abnormalities associated with these diseases, little is known about its effects in the brain and immune cells in the central nervous system. Results of this study indicate that ethanol extracts of SF (SF-E) suppress NMDA-induced reactive oxygen species (ROS) production in neurons, and LPS- and IFNγ-induced ROS and nitric oxide (NO) production in microglial cells. SF-E’s action on microglial cells appears to be mediated through inhibition of the IFNγ-induced p-ERK1/2 signaling pathway which is central to regulating a number of intracellular metabolic processes including enhancing STAT1α phosphorylation and filopodia formation. The involvement of SF in these pathways suggests the potential for novel therapeutics for stress and prevention, and/or treatment of HIV/AIDS as well as other inflammatory diseases in the brain.

## Introduction

*Sutherlandia frutescens* (SF) has long been used as a traditional medicinal plant in southern Africa for treatment of cancer, as well as a variety of chronic ailments, and more recently, HIV/AIDS [1]–[3]. Limited studies suggest multiple actions of SF as a consequence of putative antioxidant and anti-inflammatory activities [4]–[8], including inhibition of phorbol ester-induced COX-2 expression in human breast epithelial cells and mouse skin [6], [7]. There are also indications that SF has neuroprotective effects, such as alleviating symptoms associated with stress [2] as well as convulsions and epilepsy [9].

Neuroinflammation is known to play a major role in the progression of neurodegenerative diseases such as Alzheimer’s and Parkinson’s diseases, stroke, and HIV/AIDS encephalopathy [10], [11]. In many instances, activation of microglial cells, the resident macrophages in the central nervous system, is the initial step of the inflammatory response. Microglial cells can confer multiple functions, including promoting host defenses by destroying pathogens, removing debris, stimulating tissue repair, and restoring tissue homeostasis [12]. An important feature of microglial cells is their ability to undergo morphological changes enabling their rapid migration to sites of injury. Biochemically, microglial activation is associated with the release of reactive oxygen species (ROS), nitric oxide (NO), glutamate, cytokines, phospholipases and proteases [13]–[16], factors contributing to the progressive neuronal damage observed in many neurodegenerative disorders. Consequently, suppressing or limiting microglial activation can have beneficial effects for preventing neuroinflammation and neurodegeneration.

Pro-inflammatory cytokines (TNFα, IL-1β, IFNγ), and lipopolysaccharides (LPS) are commonly used to induce microglial activation *in vitro*. Several studies, including those from our laboratories, have demonstrated that pro-inflammatory cytokines induce iNOS, sPLA2-IIA and NADPH oxidase in microglia and/or astrocytes [13], [17]–[19], and these mechanisms have deleterious effects upon neurons [20]–[22]. Since a number of botanical flavonoids possess anti-inflammatory properties, there is value in assessing whether SF can offer neuroprotection by inhibiting neuroexcitatory and neuroinflammatory responses [23]. Previous studies from our laboratory have demonstrated the ability of some polyphenols to mitigate neuronal excitation otoxicity and ROS production induced by the ionotropic glutamatergic receptor agonist, N-methyl-D-aspartic acid (NMDA) [24]. Studies with microglial cells have further uncovered a mechanism involving IFNγ-induced p-ERK1/2 signaling pathways that can explain multiple modes of action of polyphenols for ameliorating oxidative and inflammatory responses [19], [25], [26]. In this study, we demonstrate that ethanol extracts of *Sutherlandia frutescens* (SF-E) mitigate NMDA-induced neuronal oxidative responses and LPS- and cytokine-induced inflammatory responses in microglial cells.

## Materials and Methods

### Materials

Dulbecco’s modified Eagle’s medium (DMEM), penicillin, streptomycin, 0.05% (w/v) trypsin/EDTA, and phosphate-buffered saline (PBS) were obtained from GIBCO (Gaithersburg, MD). Interferon-γ (IFNγ) was purchased from R & D Systems (Minneapolis, MN). Lipopolysaccharide (LPS) (rough strains) from Escherichia coli F583 (Rd mutant) and methylthiazolyldiphenyl-tetrazolium bromide (MTT) were from Sigma-Aldrich (St. Louis, MO). WST-1 kit for assay of cell viability was obtained from Clontech (Mountain View, CA). Fetal bovine serum was from Atlanta Biologicals (Lawrenceville, GA). Antibodies used for Western blots include: goat anti-rabbit IgG- horseradish peroxidase, goat anti-mouse IgG- horseradish peroxidase and anti-iNOS rabbit polyclonal (Santa Cruz Biotechnology, Santa Cruz, CA); monoclonal anti-β-actin peroxidase (Sigma-Aldrich, St. Louis, MO); STAT1α rabbit polyclonal antibody (Millipore, Billerica, MA), rabbit polyclonal p-STAT1 pSer727 (Pierce Biotechnology, Rockford, IL), rabbit polyclonal anti-ERK1/2, and mouse monoclonal anti-phospho-ERK1/2, (Cell Signaling, Beverly, MA). For ROS detection, CM-H2DCF-DA (DCF) was obtained from Invitrogen, Inc. (Eugene, OR), and dihydroethidium (DHE) from Sigma-Aldrich (St. Louis, MO).

### *Sutherlandia Frutescens*

Freeze-dried milled vegetative parts of SF were purchased from Big Tree Nutraceutical (Fish Hoek, South Africa). This product was stored at −20°C in an air-tight container in the dark, and as required, samples (50 g) were extracted with 500 mL of ethanol at room temperature on a rotating shaker. The sample was vacuum-filtered and the solids were returned to the flask and twice more extracted with ethanol while agitating. The combined filtrates were evaporated to dryness under a vacuum. SF ethanolic extracts (SF-E) were weighed and re-suspended in DMSO prior to use in cell culture. No change in response of SF extract on LPS+IFNγ-induced NO production upon storage of the extract at −20°C for 30 days was observed (data not shown).

### Cell Culture

#### Primary rat cortical neurons

All animal care and experimental protocols were carried out in accordance with NIH guidelines and with permission from the University of Missouri Animal Care and Use Committee (protocol #6728). Primary cortical neurons were prepared from fetal brain of Sprague–Dawley rats (Harlan Laboratories, Indianapolis, IN) at E17 (embryonic day 17) using protocols described previously [24], [26] with slight modifications. In brief, cerebral cortices were dissected in Hanks buffer followed by incubation with 0.05% trypsin (GIBCO, Grand Island, NY) at 37°C for 45 min. After dispersing the cortices with a pasture pipette, suspension was centrifuged at 2000×g for 10 min. The cell pellet was re-suspended in D10C medium (DMEM including 10% BCS, 10% Ham’s F-12 medium, 2 mM l-glutamine, 2.5% Hepes and 0.25% Pen/Strep). Cells (1.3×10^5^/cm^2^) were seeded into 50 mg/L poly-L-lysine-coated dishes in D10C medium. After 4–5 h, the D10C medium was replaced with Neurobasal medium containing 2% B27-AO, 2 mM L-glutamine and 1% Pen/Strep. Cells were cultured at 37°C in a humidified incubator with 5% CO_2_ for at least 8 days before experiments, and half of the medium was replaced with fresh medium every 3–4 days. Immunostaining for specific markers of astrocytes (GFAP, glial fibrillary acidic protein), microglia (OX-42) and neurons (MAP-2, microtubule-associated protein-2) indicated that the 8-day-old neuronal cultures contained only 3–4% astrocytes and 3% microglia [24]. Neuronal morphology was routinely visualized using an inverted microscope from Olympus (Center Valley, PA) with a 20× objective.

#### Microglial cells

The immortalized mouse (BV-2) cells were originally obtained from Dr. R. Donato (University of Perugia, Italy) [18]. The immortalized rat microglial cell line HAPI was a gift from Dr. J. Hong (Laboratory of Toxicology and Pharmacology, National Institute of Environmental Health Sciences, National Institute of Health, Research Triangle Park, NC) [19]. Both BV-2 and HAPI microglial cells were cultured as described previously [19]. Briefly, cells were cultured in 75 cm^2^ flasks with DMEM (high glucose) supplemented with 10% FBS containing 100 units/mL penicillin and 100 µg/mL streptomycin, and maintained in 5% CO_2_ incubator at 37°C. For subculture, cells were removed from the culture flask by gentle scraping, re-suspended in the culture medium and sub-cultured in 6/96-well plates for experiments. Cells were serum starved for 4 h prior to adding cytokines and LPS. SF-E was added 1 h before LPS and/or IFNγ treatment. Cell morphology was observed by using a phase contrast Nikon DIAPHOT 300 microscope attached with a CCD cool camera, and a MagnaFire 2.1C software was used for image capture and processing. Representative bright field pictures were obtained using a 20× objective.

### Assessing Cell Viability

In this study, we used MTT assay protocol to test whether SF-E alter the oxido-reductase activity in live neurons. Briefly, neurons were treated with specified concentrations of SF-E for 24 h. After incubation, the medium was removed and 100 µL of MTT reagent (0.5 mg/mL) dissolved in DMEM was added to each well. The plates were incubated for 3 h at 37°C, and the formazan particles formed by reduction of the tetrazolium dye to its insoluble formazan were dissolved with 100 µL DMSO in each well, and absorbance at 540 nm was measured with a microplate reader (Biotek Synergy 2, Winooski, VT).

For microglial cells, we used a protocol containing WST1 (Water Soluble Tetrazolium salt) (Clontech, Mountain View, CA) to determine effects of SF-E on cell viability. WST1 has advantage over MTT because it yields a water soluble formazan and can be read directly without the solubilization procedure. Briefly, cells treated with specified concentrations of SF-E were incubated for 16 h. After incubation, the medium was removed and 10 µL of WST1 reagent (1∶10 of premixed reagent) dissolved in 90 µL DMEM was added to each well. The plates were incubated for 30 min at 37°C and absorbance of formazan produced by the dehydrogenase activity of the living cells was measured at 450 nm using a microplate reader (Biotek Synergy 2, Winooski, VT).

### Nitric Oxide (NO) Determination

NO released from cells was converted to nitrite in the culture medium and determined using the Griess reagent protocol [25]. In brief, cells in 96-well plate were serum-starved in phenol red-free DMEM for 3 h, followed by incubation with specified concentrations of SF-E or inhibitors for 1 h, and then treated with IFNγ and/or LPS at 37°C for 16 h. Aliquots of the media (50 µL) were incubated with 50 µL of the reagent A (1% (w/v) sulfanilamide in 5% phosphoric acid, Sigma-Aldrich) for 10 minutes at room temperature covered in dark. This was followed by incubation with 50 µL of reagent B (0.1%, w/v, N-1-napthylethylenediamine dihydrochloride, Sigma-Aldrich) for 10 minutes at room temperature, protected from light, and A_543_ nm was measured using a microplate reader. Serial dilutions of sodium nitrite (0–100 µM) were used to generate the nitrite standard curve.

### ROS Determination

ROS production in neurons was measured with dihydroethidium (DHE) [26]. In brief, neurons were cultured on 35 mm dishes pre-coated with poly-L-lysine. After treating with SF-E for 30 min, cells were then exposed to 100 µM NMDA for 30 min in phenol red-free Neurobasal medium with 0.5 mg/mL BSA. At 30 min prior to image acquisition, cells were loaded with 10 µM DHE and incubated at 37°C. Fluorescence images were acquired using a Nikon TE-2000 U inverted microscope with a 20× NA 0.95 objective and a cooled CCD camera controlled with the MetaView imaging software (Universal Imaging, West Chester, PA). For each field, the total fluorescence was measured and normalized by the total number of cells. For each treatment group, at least three random images from the same dish were captured and analyzed, and each treatment was repeated three times independently for statistical analysis.

ROS production in microglial cells was based on the protocol using CM-H2DCF-DA [25]. Microglial cells were seeded in 96-well plate and grown until 90% confluent. They were serum-starved for 3 h, followed by pretreatment with SF-E or inhibitors for 1 h prior to stimulation with LPS and IFNγ for 11 h. CM-H2DCF-DA (10 µM) was added to each well and further incubated for 1 h. The fluorescent intensity of DCF was measured with a Synergy4 microplate reader (excitation wavelength of 490 nm and emission wavelength of 520 nm).

### Western Blot Analysis

Cells were harvested in RIPA buffer containing 50 mM Tris-HCl (pH 7.5), 150 mM NaCl, 1% Nonidet P-40, 0.5% sodium deoxycholate, and 0.1% SDS. The extract was centrifuged at 10,000×g for 15 min at 4°C to remove cell debris. Protein concentration was determined with the BCA protein assay kit (Pierce Biotechnology, Rockford, IL). For each sample, 5 µg of protein was loaded and resolved in SDS-PAGE and run at 100 V. After electrophoresis, proteins were transferred to 0.45 µm nitrocellulose membranes at 100 V for 1.5 h. Membranes were blocked in Tris-buffered saline (TBS), pH 7.4, with 0.1% Tween 20 (TBS-T) containing 5% non-fat milk for 1.5 h at room temperature. For different experiments, the blots were incubated with ERK1/2 (1∶2000), phospho-ERK1/2 (1∶1000), iNOS polyclonal (1∶1000) antibodies, STAT1α polyclonal antibodies (1∶1000), and p-STAT1 pSer727 polyclonal antibodies (1∶1000) overnight at 4°C. After repeated washing with 1X TBS-T, blots were incubated with goat anti-rabbit IgG-HRP (1∶5000) or goat anti-mouse IgG-HRP (1∶2000) for 1 h at room temperature. The blots were then washed three times with 1X TBS-T. Immuno-labeling was detected by chemiluminescence ECL/WestPico/Femto. For loading control, blots were incubated with monoclonal anti-β-actin peroxidase (1∶30,000). Blots were scanned and the optical density of protein bands was measured using the QuantityOne program (BioRad, Hercules, CA).

### Assessment of Filopodia

Cell morphology was observed by using a phase contrast Nikon DIAPHOT 300 microscope with a 20× objective and a CCD cool camera attached to MagnaFire 2.1C software for image capture/processing. Representative bright field pictures were captured with 20× objective and filopodia in cells were counted manually by persons blinded to the treatment groups. Results were expressed as % cells with filopodia relative to the total number of cells in each field [19].

### Statistical Analysis

Data are presented as means ± SEM. Results were analyzed either by one-way ANOVA followed by Dunnett’s multiple comparison tests or two-way ANOVA with Bonferroni post-tests (V4.00; GraphPad Prism Software Inc., San Diego, CA). Statistical significance was considered for p<0.05.

## Results

**Figure 1:**
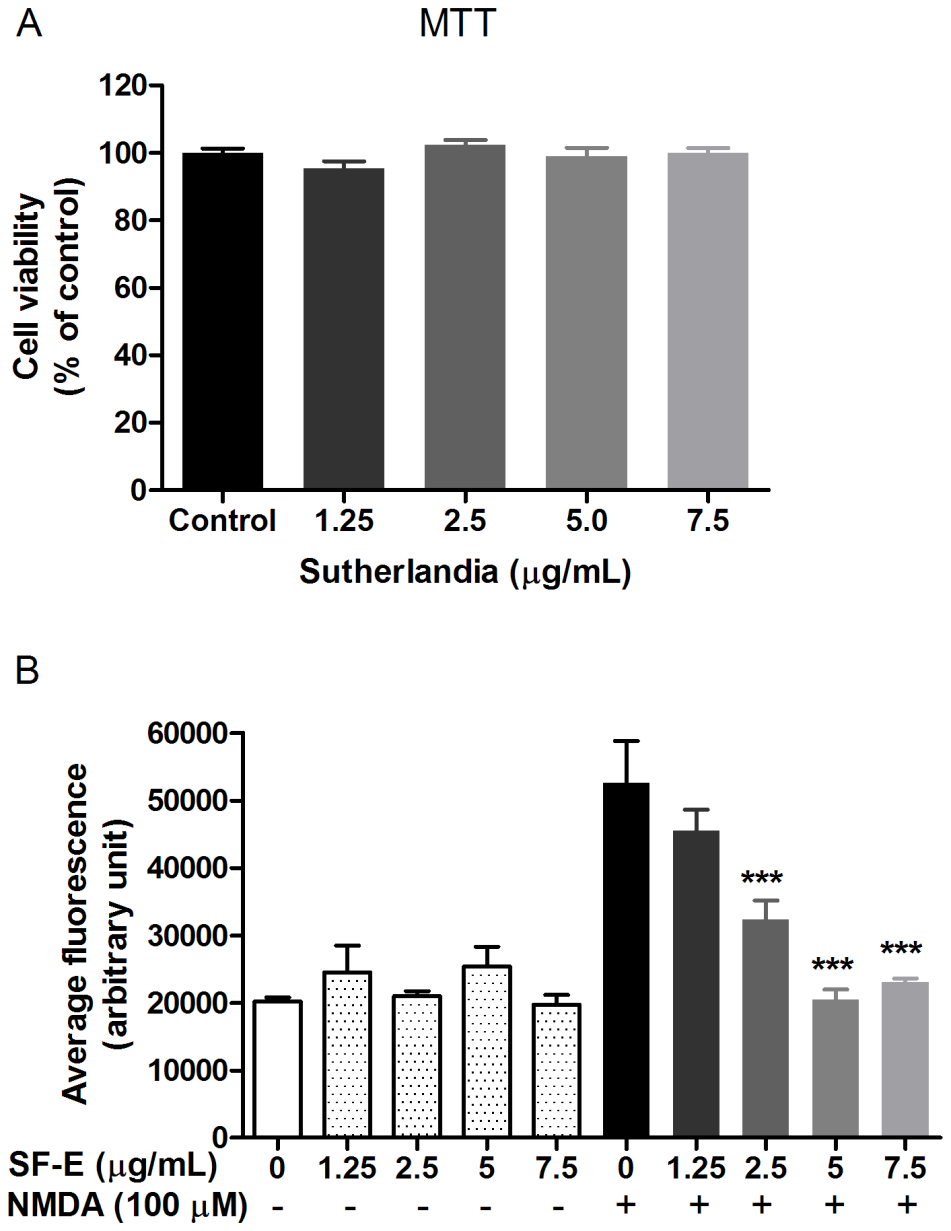
SF-E inhibits NMDA-induced ROS production without altering cell viability of primary cortical neurons. (A) Exposure of SF-E (0–7.5 µg/mL) to primary cortical neurons for 24 h did not alter neuronal viability as assayed by MTT. Data are expressed as the mean ± SEM from 3 individual experiments and analyzed by one-way ANOVA (p = 0.1774). (B) Bar graph of average fluorescence, depicting inhibition of NMDA-induced ROS production by SF-E. ROS production was determined in primary neurons after treating cells with SF-E (0–7.5 µg/mL) for 30 min prior to stimulation with NMDA (100 µM) for 30 min. For ROS production, neurons were loaded with dihydroethidium (DHE, 10 µM) 30 min prior to image acquisition. Data are expressed as the mean ± SEM from 3 individual experiments and analyzed by two-way ANOVA with Bonferroni post-tests. ***indicates significant decrease in ROS production by SF-E as compared to NMDA (p<0.001).

Based on earlier work indicating that NMDA, the ionotropic glutamatergic receptor agonist, stimulates rapid production of ROS in neurons through activation of NADPH oxidase [19], several studies have demonstrated that botanical polyphenols such as EGCG from green tea as well as honokiol and magnolol from magnolia bark suppress this ROS pathway [25], [26]. In this study, exposure of SF-E to primary rat cortical neurons for 24 h did not alter MTT oxido-reductase activity, frequently used to represent neuronal viability (Fig. 1a). However, when neurons were first exposed to SF-E for 30 min and followed by stimulation with NMDA, results indicated ability for SF-E to inhibit NMDA-induced ROS production in a dose-dependent manner, with a maximum inhibition at 5 µg/mL (Fig. 1b).

**Figure 2:**
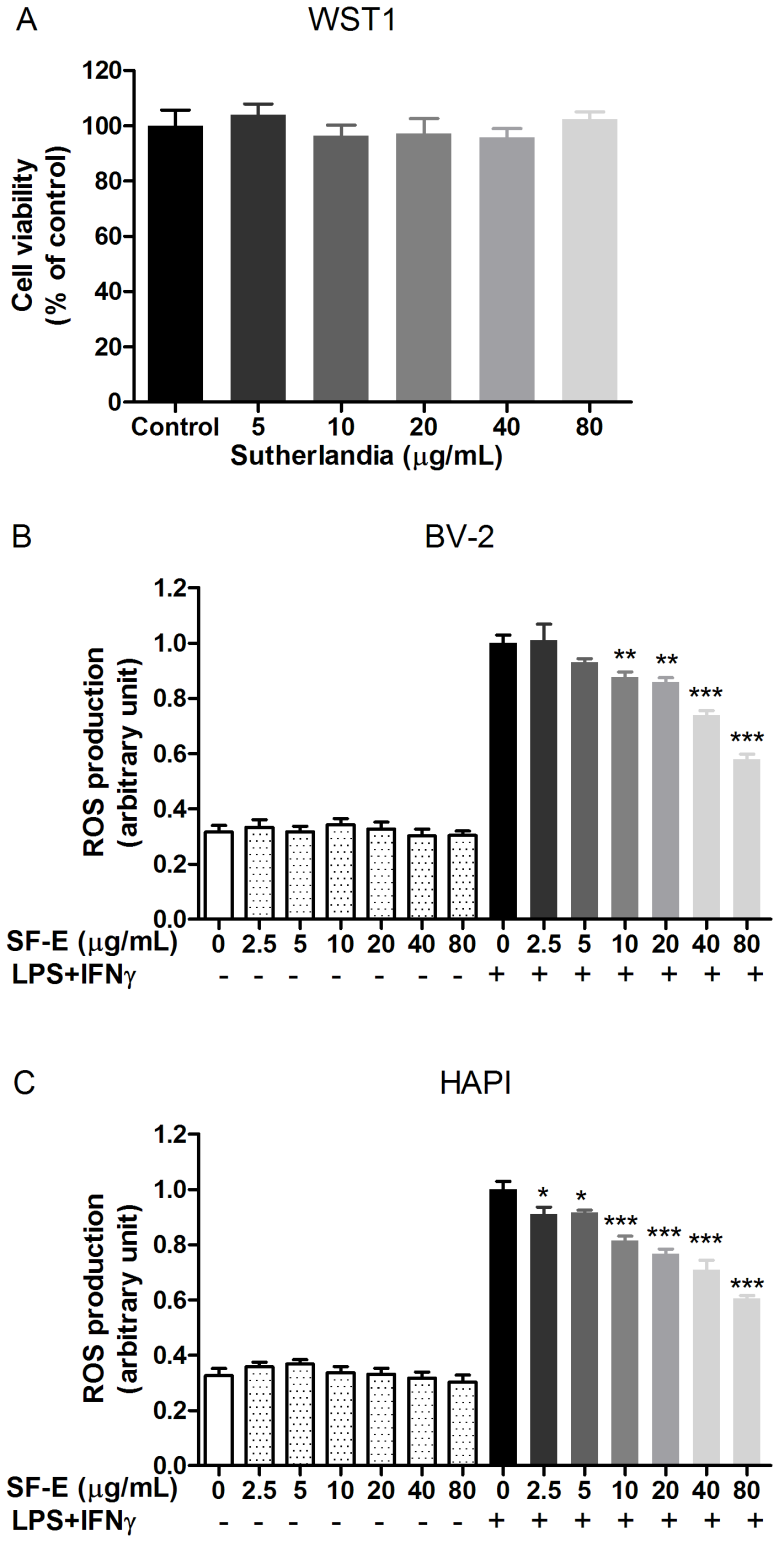
SF-E effects on microglial cell viability and ROS production. (A) Exposure of SF-E (0 to 80 µg/mL) to BV-2 microglial cells for 16 h did not alter cell viability as assayed by WST1. Data are expressed as the mean ± SEM from 3 individual experiments and analyzed by one-way ANOVA (p = 0.6828). SF-E inhibits LPS+IFNγ-induced ROS production in BV-2 (B) and HAPI (C) microglial cells**.** SF-E (0 to 80 µg/mL) were applied to cells 1 h prior to exposure to a combination of LPS (100 ng/mL) and IFNγ (10 ng/mL) for 12 h. ROS production was measured using CM-H2DCFDA as described in the text. Results are expressed as the mean ± SEM (n = 3) and analyzed by two-way ANOVA with Bonferroni post-tests. *p<0.05; **p<0.01; ***p<0.001 as compared to the respective LPS+IFNγ-stimulated group.

Similar to neurons, SF-E did not alter oxido-reductase activity in microglial cells (Fig. 2a). Unlike neurons which showed ROS production upon short time stimulation with NMDA, ROS induced by LPS+IFNγ in microglial cells followed a delayed time course, starting from 4 h and peaking at 12 h [25]. In this study, SF-E caused a dose-dependent decrease in ROS production by microglial cells treated with LPS+IFNγ for 12 h (Fig. 2b). Comparing with NMDA-induced ROS production in neurons, much higher levels of SF-E were required for inhibition of ROS production in microglial cells (Fig. 1b vs. Fig. 2b). Similarly, SF-E also decreased LPS+IFNγ-induced ROS production in HAPI cells, an immortalized microglial cell line from rats (Fig. 2c). In both neurons and microglial cells, exposure of cells with SF-E alone did not alter basal levels of ROS (Fig. 1b, Fig. 2b and 2c).

**Figure 3:**
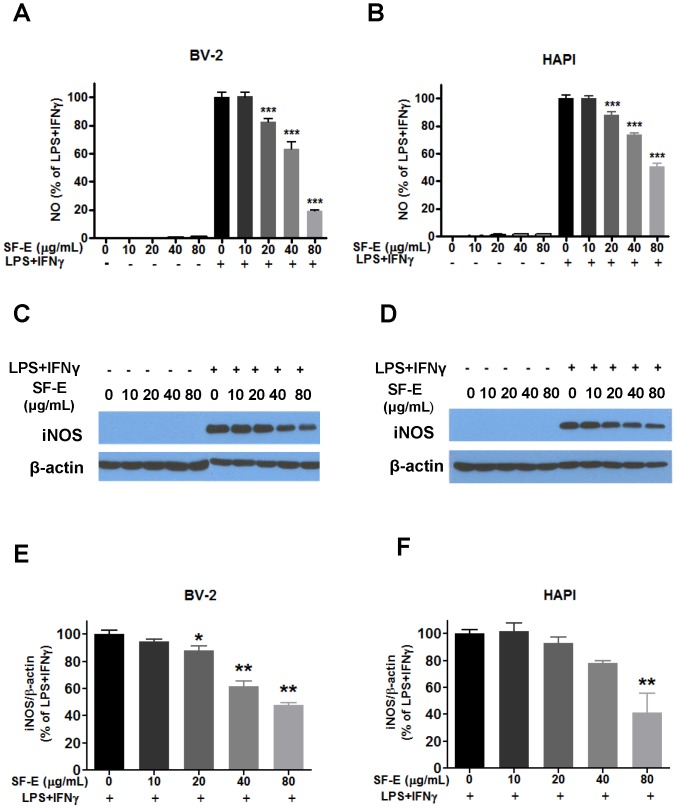
SF-E inhibits LPS+IFNγ-induced NO production and iNOS expression in microglial cells. (A-B) Cells were treated with SF-E (0 to 80 µg/mL) for 1 h followed by stimulation with LPS (100 ng/mL) and IFNγ (10 ng/mL) for 16 h. Culture media were collected for determination of NO using the Griess reaction protocol as described in the text. (C-D) Representative Western blots depicting LPS+IFNγ-induced iNOS protein expression in BV-2 and HAPI microglial cells incubated in the presence and absence of SF-E. (E-F) Bar graphs representing iNOS/β-actin ratios using LPS and IFNγ as control (100%). Results are expressed as the mean ± SEM (n = 7) and significant differences from the respective LPS+IFNγ stimulated group was determined by one-way ANOVA followed by Dunnett’s tests, *p<0.05; **p<0.01.

LPS- and IFNγ-induction of iNOS and production of NO are important inflammatory responses of microglial cells. In this study, no NO production was observed in BV-2 or HAPI microglial cells after treatment with SF-E alone (Fig. 3a and 3b). However, a dose-dependent decrease in NO production was observed upon treating cells with SF-E after LPS+ IFNγ, with significant inhibition starting at 20 µg/mL SF-E (Fig. 3a and Fig 3b). SF-E was a more effective inhibitor in BV-2 cells than in HAPI cells (Fig. 3a vs. 3b). Western blot analysis also showed a corresponding decrease in iNOS protein expression with increasing levels of the SF-E added to both BV-2 and HAPI cells (Fig. 3c and 3d). Corresponding to NO production, SF-E was more effective in inhibiting iNOS protein production in BV-2 cells as compared to HAPI cells (Fig. 3e and 3f).

**Figure 4:**
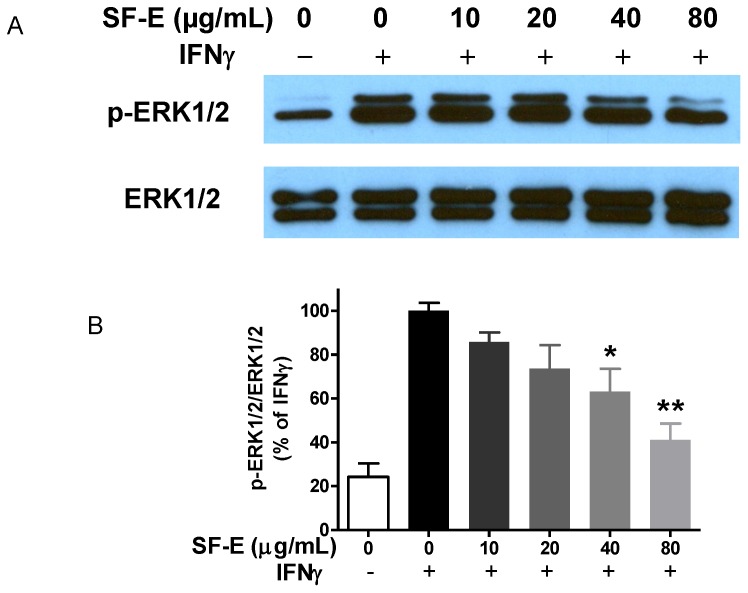
SF-E inhibits IFNγ-induced activation of ERK1/2 in BV-2 microglial cells. (A) Western blot analysis showing a representative experiment of SF-E pretreatment on IFNγ-induced ERK1/2 phosphorylation in BV-2 microglial cells. Cells were treated with SF-E (0 to 80 µg/mL) for 1 h followed by stimulation with IFNγ (10 ng/mL) for 8 h. (B) Bar graphs representing p-ERK1/2/ERK1/2 ratios using IFNγ as control (100%). Results are expressed as the mean ± SEM (n = 5) and were analyzed by one-way ANOVA followed by Dunnett’s tests, *p<0.05; **p<0.01.

**Figure 5:**
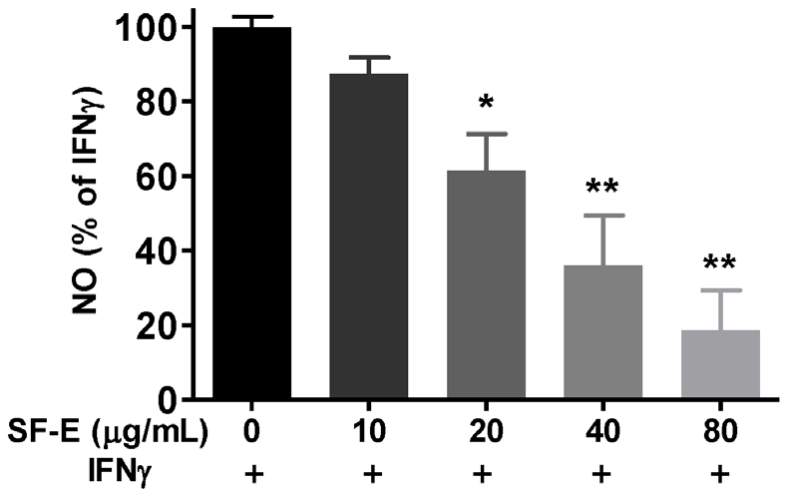
SF-E inhibits IFNγ-induced NO production in BV-2 microglial cells. Cells were treated with SF-E (0 to 80 µg/mL) for 1 h followed by stimulation with IFNγ (10 ng/mL) for 16 h. Culture media were collected for determination of NO using the Griess reaction protocol as described in the text. Results are expressed as the mean ± SEM (n = 3) and significant difference from the IFNγ-stimulated group was determined by one-way ANOVA followed by Dunnett’s tests, *p<0.05, **p<0.01.

**Figure 6:**
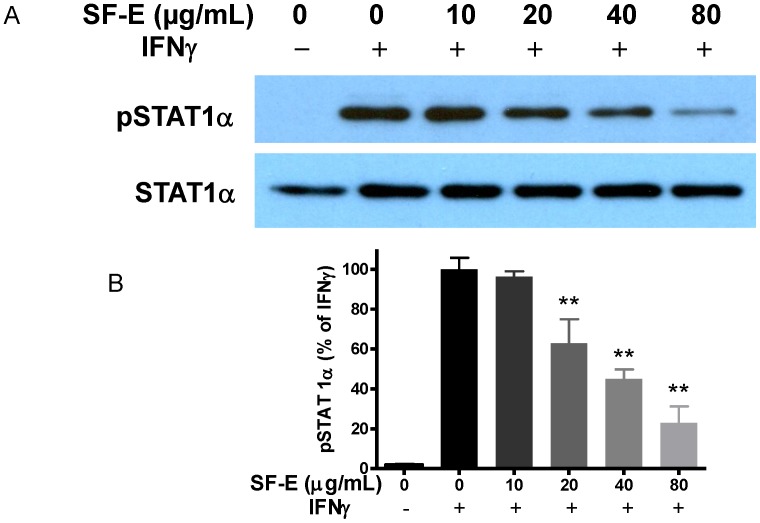
SF-E inhibits IFNγ-induced phosphorylation of STAT-1α. (A) Western blot analysis showing a representative experiment of SF-E pretreatment on IFNγ-induced STAT1α phosphorylation in BV-2 microglial cells. Cells were treated with SF-E (0 to 80 µg/mL) for 1 h followed by stimulation with IFNγ (10 ng/mL) for 8 h. (B) Bar graphs representing p-STAT1α/STAT1α ratios using IFNγ as control (100%). Results are expressed as the mean ± SEM (n = 3) and significant difference from the IFNγ-stimulated group was determined by one-way ANOVA followed by Dunnett’s tests, **p<0.01.

Although induction of iNOS typically requires transcription factors from the NF-κB and JAK-STAT pathways, our previous study showed that in BV-2 and HAPI microglial cells, LPS and IFNγ can independently induce iNOS and NO [19], thus suggesting a cross-talk mechanism between the JAK-STAT and NF-κB pathways. In search of this cross-talk mechanism, we discovered that IFNγ induced a MAPK pathway leading to phosphorylation of ERK1/2 [25]. Subsequently, phosphorylation of ERK1/2 led to stimulate multiple metabolic reactions including production of ROS by NADPH oxidase and induction of iNOS/NO [25]. In this study, we investigated whether SF-E’s action on microglial cells is also mediated through targeting the IFNγ-induced p-ERK1/2 pathway. As shown in Fig. 4a and 4b, SF-E showed a dose-dependent decrease in IFNγ-induced p-ERK1/2 expression. Furthermore, SF-E also inhibited IFNγ-induced NO production (Fig. 5) and phosphorylation of STAT1α (Fig. 6).

**Figure 7:**
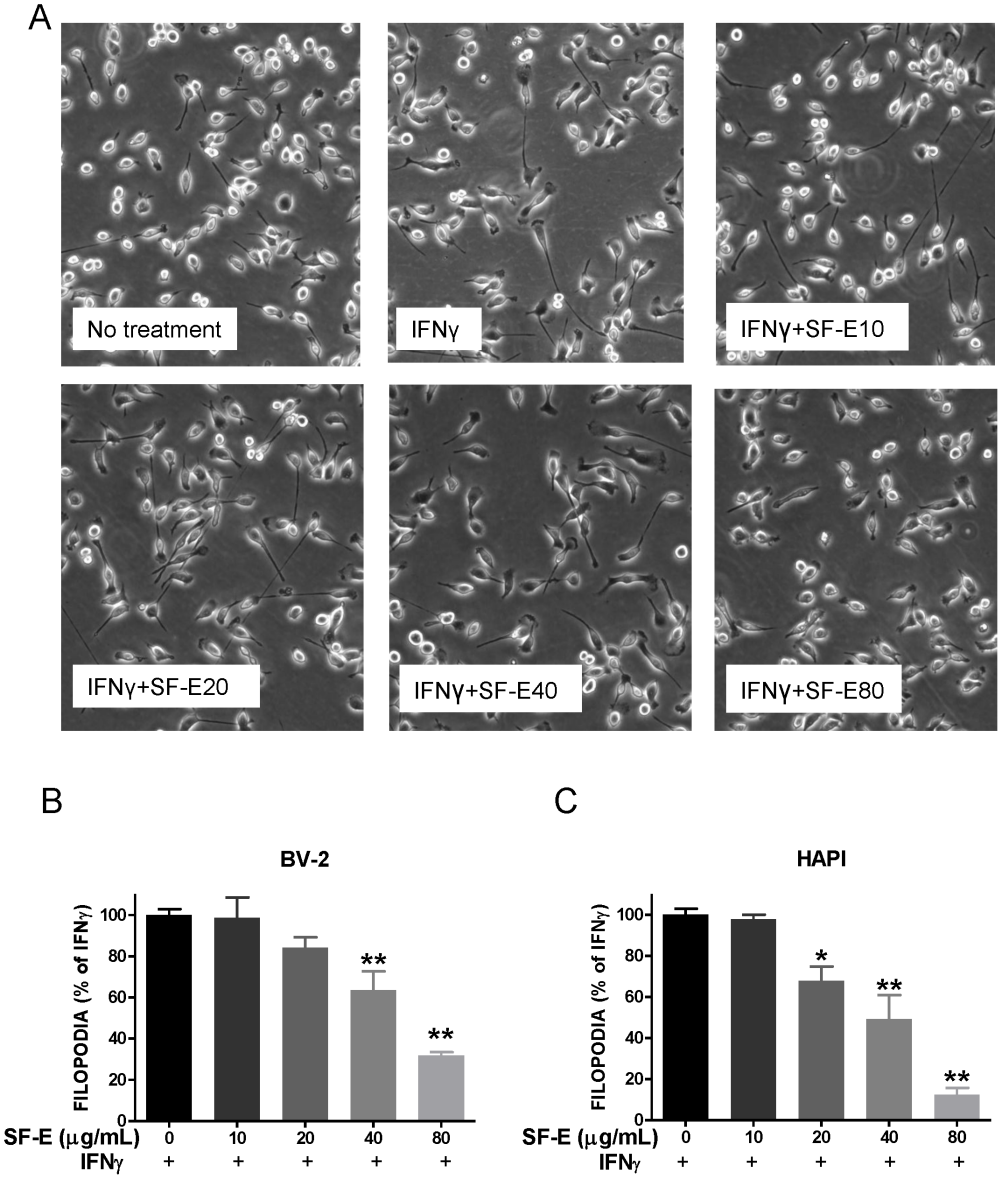
SF-E inhibits IFNγ-induced filopodia production in microglial cells. (A) Representative bright field photomicrographs of BV-2 cells were taken with an inverted Nikon microscope (20× objective). (B, C) BV-2 and HAPI cells were pretreated with SF-E (0–80 (µg/mL) for 1 h prior to exposure to IFNγ (10 ng/mL) for 4 h. Determination of cells containing filopodia at 4 h after exposure to IFNγ. Results are expressed as % of filopodia containing cells versus total cell numbers using the IFNγ-stimulated group as control, 100% (see Methods). Results are expressed as the mean ± SEM (n = 3–4) and significant differences from the respective IFNγ-stimulated group was determined by one-way ANOVA followed by Dunnett’s tests, *p<0.05; **p<0.01.

A characteristic property of microglial cells is their ability to proliferate and change shape under different stages of activation. In our earlier study, we observed that IFNγ induced an increase in filopodia production in microglial cells (maximum at 4 h) and the induction could be abolished by U0126, a specific inhibitor for MEK1/2, the kinases responsible for phosphorylation of ERK1/2 [19]. In this study, morphological examination of microglial cells indicated that IFNγ induced a two-fold increase in filopodia in BV-2 microglial cells (13.2±1.0 in controls versus 27.1±2.0 in IFNγ-stimulated cells). Pretreatment with SF-E inhibited IFNγ-induced filopodia formation in BV-2 microglial cells (Fig. 7a and 7b). Similarly, SF-E elicited a dose-dependent decrease in filopodia in HAPI cells (Fig. 7c).

## Discussion

SF has been recognized for its broad medicinal uses but the mechanisms for mediating the claimed effects associated with the central nervous system are not well understood [3]. Although previous studies have provided evidence for SF’s ability to alleviate symptoms associated with stress [2] and childhood convulsions and epilepsy [9], it is not known whether these effects are associated with ability for this herb to offer anti-oxidative and anti-inflammatory properties. Recent studies have demonstrated an important role of microglial cells in mediating neuroinflammatory responses in a number of neurological disorders [13], [20], [27]. In particular, HIV-associated neurocognitive disorders (HAND), including HIV-associated dementia (HAD), as well as HIV encephalopathy and mild neurocognitive disorders (MND) are important and prevalent in people living with HIV/AIDS [28], [29]. In sub-Saharan Africa, the nexus of the HIV/AIDS pandemic, Sutherlandia is widely used to treat conditions associated with HIV/AIDS infection [29]. This study is the first to investigate the anti-oxidative and anti-inflammatory responses of SF on neurons and microglial cells. Ability to mitigate these responses may shed new light on how SF exerts its neuroprotective effects in people living with HIV/AIDS.

The ionotropic glutamatergic receptor agonist, such as NMDA, is known to cause rapid and massive calcium influx to neurons, and subsequently leading to activation of calcium-dependent kinases, caspases, mitochondrial dysfunctions, and apoptotic cell death. In addition, NMDA-induced neuronal excitation is linked to ROS production through activation of NADPH oxidase [24], [30]. The increase in neuronal ROS production can be suppressed by botanical antioxidants, such as EGCG from green tea [26] and honokiol and magnolol from Magnolia bark [25]. In this study, SF-E could similarly inhibit NMDA-induced ROS production. In agreement with the notion that neurons are more sensitive to oxidative stress than microglial cells, the levels for SF-E required to inhibit NMDA-induced ROS in neurons were 5 times lower than those required for inhibition of ROS production due to LPS+IFNγ in microglial cells. On the other hand, since ROS induced by NMDA in neurons is more rapid than those in microglial cells, more studies are needed to determine whether mitigation of neuronal ROS by SF-E or other botanical antioxidants may effectively suppress the down-stream events leading to neuronal apoptosis.

Results in our previous studies have demonstrated that besides the canonical JAK-STAT transcriptional pathway, IFNγ could also activate protein kinases including PKC and ERK1/2 [25], [31]. Other studies further demonstrated the role of p-ERK1/2 in activating cellular metabolism including phosphorylation of p47phox, an important cytosolic subunit of NADPH oxidase for ROS production, phosphorylation of STAT1α [32], as well as proteins for induction of filopodia [19]. Similar to the action of ginsenoside Rh1 [33], results here also show that SF-E can suppress iNOS via the JAK/STAT and ERK1/2 signaling pathways in microglial cells. The ability of SF-E to inhibit IFNγ-induced p-ERK1/2 and subsequently ROS and NO production can explain its actions on multiple oxidative and inflammatory responses in microglial cells.

SF is known to contain a number of components which might be responsible for its pleiotropic activity [3], [34]. The presence of GABA, an inhibitory neurotransmitter, may contribute to control of childhood convulsions and epilepsy [9]. L-canavanine also is of interest, since earlier studies showed that this compound could suppress NO accumulation in astrocytes [35]. Furthermore, the presence of triterpenes could account for its effects upon adrenocorticosteroid metabolism, and reduction of corticosterone levels and treatment of anxiety and stress [2]. Faleschini et al. [34] further indicated modest effects of SF ethanol extracts for induction of IL-8 and TNFα by phorbol myristoyl acetate (PMA), a compound known to activate protein kinase C and related pathways. Studies with other systems also demonstrated the ability for SF to promote anti-inflammatory and wound healing activities by inhibiting expression of COX-2 induced by phorbol ester in epithelial cells [6], [7]. In preliminary studies attempting to determine whether a single class of compounds is responsible for the observed effects upon microglial cells and neurons, we have not been able to demonstrate activity of the triterpene fractions (unpublished results). SF also contains unique flavonol glycosides, and based upon our previous work [36], these are good candidates for the observed inhibitory effectors. Although further studies are needed, these flavonol glycosides may offer similar action as curcumin, a polyphenol from the curry spice, which was shown to mitigate HIV-1 gp120-mediated inflammation and apoptosis in primary neurons and in microglial cells [37].

## Conclusion

This study demonstrates the ability of SF-E to inhibit NMDA-stimulated ROS in neurons and LPS- and IFNγ-stimulated ROS and iNOS/NO production in microglial cells. As SF-E can also inhibit p-ERK1/2 and multiple kinase activities, these results offer an explanation for the multi-mode of action of SF and its potential use for prevention and/or treatment of inflammatory illnesses including HIV-associated neurocognitive disorders (HAND).
